# Ischemic duration determines extent of cardiac remodeling, and both early and delayed reperfusion prevent fatal cardiac rupture: Model comparison

**DOI:** 10.1371/journal.pone.0328001

**Published:** 2025-08-22

**Authors:** Ling Zhao, Amanguli Ruze, Guo-Li Du, Min-Tao Gai, Jing Tang, Xiao-Ming Gao

**Affiliations:** 1 Department of Cardiology, State Key Laboratory of Pathogenesis, Prevention and Treatment of High Incidence Diseases in Central Asian, First Affiliated Hospital, Clinical Medical Research Institute of Xinjiang Medical University, Urumqi, China; 2 Xinjiang Key Laboratory of Medical Animal Model Research, Urumqi, China; 3 Department of Endocrinology, First Affiliated Hospital of Xinjiang Medical University, Urumqi, China; 4 Xinjiang Key Laboratory of Cardiovascular Disease, Urumqi, China; 5 Department of Clinical Laboratory, First Affiliated Hospital of Xinjiang Medical University, Urumqi, China; National Institutes of Health, UNITED STATES OF AMERICA

## Abstract

High incidence of cardiac rupture in murine myocardial infarction (MI) model leads to a substantial loss before the study end-point. Selecting animal models with varying degrees of injury for different research purposes is crucial for cardiovascular research. Male C57 mice were subjected to ischemia/reperfusion (I/R) or permanent occlusion (MI) injury. The incidence of cardiac rupture, degree of myocardial injury, inflammatory responses, left ventricular (LV) remodeling and infarct myocardium healing were examined. Compared to MI mice, early reperfusion (1, 2 and 4h I/R) completely prevented cardiac rupture, while delayed reperfusion (12h and 24h I/R) significantly reduced incidence of cardiac rupture to 5.7% and 8.6%, respectively. In the acute phase, prolonged ischemia increased infarct size, myocyte apoptosis, and both systemic and regional inflammatory responses. These changes correspond to enhanced MMP-9 activity and a weakening of the tensile strength of the infarcted myocardium. Following ischemic insult, early reperfusion was associated with less extent of myocardial injury, inflammatory response and adverse cardiac remodeling, whereas, delayed reperfusion and MI groups exhibited severe myocardial damage and remodeling. Furthermore, both early and delayed reperfusion were associated with increased infiltration of type 2 macrophages and proliferation of endothelial cells during the early healing phase, thereby facilitating healing of the infarct myocardium. Delayed reperfusion resulted in a comparable and substantial degree of cardiac remodeling but with a lower risk of cardiac rupture in comparison with MI model. This feature makes it a feasible model for cardiac ischemia research.

## Introduction

Ischemic heart disease has a high prevalence and is the most common cause of death worldwide. Cardiac rupture is a catastrophic complication of acute myocardial infarction (MI), with an incidence rate of 1.3% in acute coronary syndromes (ACS) patients [1]. Early coronary recanalization leads to myocardial salvage, preservation of global left ventricular (LV) function and improvement of survival and long-term outcomes [2–4]. Timely reperfusion therapies, including thrombolysis, primary coronary intervention or coronary artery bypass grafting, are the primary treatments for acute MI. These therapies are also believed to prevent cardiac rupture, an extreme form of acute remodeling, in most patients [5]. However, substantial patients cannot receive early reperfusion therapy because of regional medical care limitation, poor emergent transport availability and late presentation which leads to worse clinical outcomes [6]. Implementation of delayed reperfusion therapy is a key issue in clinical practice for the management of acute MI.

In this context, animal models for ischemic heart disease play a crucial role in understanding the pathophysiology and exploring potential therapeutic strategies. Currently, animal models for ischemic heart disease mainly include MI model induced by permanent occlusion of the left coronary artery and ischemia/reperfusion (I/R) model induced by certain time of the left coronary artery occlusion following release the occlusion to achieve reperfusion. While these two models have been widely used to study pathophysiological changes, molecular mechanisms, and therapeutic effects, they also have significant limitations. For example, MI model often achieves a large infarct size, devastating pathological remodeling and progressive heart failure [7]. Meanwhile, the MI model causes a higher incidence of fatal cardiac rupture, leading to significant early animal loss. This complicates long-term studies and increases the burden on researchers by necessitating larger group sizes and additional procedures and costs [8]. I/R model better mimics the clinical situation of human patients receiving reperfusion therapies. However, the occlusion (ischemia) time before reperfusion varies widely, ranging from 30 min to 1, 2 or 4 h in most published studies [9,10]. Consequently, the resulting infarct sizes (% of LV) fluctuate significantly, from 12% to 60% [10–12], compared to the more consistent and larger infarct size (35%−50%) from MI model [13,14]. An I/R model with short-ischemic duration causes a lesser extent of myocardial necrosis and cardiac remodeling, limiting the scope for investigating novel interventions. Additionally, since the “symptom-to-balloon” time often exceeds 1 hour in many countries [15], short-ischemia models may not accurately reflect real-world clinical scenarios.

To address these limitations, we conducted a study to examine the effects of reperfusion after varying periods of myocardial ischemia on the prevention of cardiac rupture and its impact on myocardial pathology. Furthermore, we delved into the underlying mechanisms by which reperfusion can mitigate fatal cardiac rupture. The aim of our study is to find a suitable small rodent model of cardiac ischemia to meet the diverse needs of medical research.

## Methods and materials

### Animals

Male C57B/6J mice aged 10–12 weeks were used in this study. C57BL/6J mice were purchased from Weitong-Lihua Company (Beijing, China). Studies related to mice were all approved by the Ethics Committee of the First Affiliated Hospital of Xinjiang Medical University (No. 20180223-92), and followed the relevant guidelines. The reporting of animal experiments follows recommendations in the ARRIVE guidelines. All methods were performed in accordance with relevant guidelines/regulations.

### Establishment of MI and I/R models

Animals were anesthetized using a combination of ketamine, xylazine, and atropine (KXA, 100, 20 and 1.2 mg/kg, respectively) via intraperitoneal (i.p.) injection. Mice were then orally intubated and mechanically ventilated. Prior to surgery, mice were anesthetized and carprofen (5 mg/kg, 0.5 mg/ml, Pfizer New Zealand Pty Ltd) was administered subcutaneously (s.c.) for analgesia [16]. Mice were then administered a local analgesic (lignocaine, 2 mg/kg, 2 mg/ml s.c.) at the site of incision [16]. Open-chest surgery was performed to induce MI model by permanent occluding LAD or to induce ischemia-reperfusion (I/R) model by occluding left anterior descending coronary artery (LAD) for 1, 2, 4, 12 and 24 h followed by re-opening the occlusion for certain periods of reperfusion (for different purposes), respectively, as previously described [7,17,18]. Sham operation was also performed without occluding the LAD. All surgical procedures were carried out with the surgeon blinded to the group information.

### Autopsy, tissue collection and organ weight

All mice subjected to surgery were closely monitored during the study period, autopsy was performed in all mice found died to identify the cause of death such as cardiac rupture or heart failure. The presence of a large amount of blood clot around the heart and in the chest cavity as well as a perforation of the infarcted wall indicated rupture death. The cause of death was attributed to acute heart failure in mice exhibiting all of the following conditions: the presence of an infarct, pulmonary congestion (increased lung wet weight), and massive chest fluid accumulation, as described previously [7]. On day 3, some mice were deeply anesthetized via isoflurane inhalation and euthanized by exsanguination to collect blood and heart tissue. Following echocardiography at the last time-point, the remaining surviving mice were deeply anesthetized and euthanized by cervical dislocation at week 4 for tissue collection, the heart, lungs and tibia were harvested and weighed or measured.

### Infarct size measurement

Some mice from each ischemic group were deeply anesthetized with isoflurane and euthanized by exsanguination at 48 h after I/R or MI to determine the infarct size. A dual staining method with 5% Evans Blue and 1.5% triphenyltetrazolium (TTC) was used, as described previously [11].

### Quantitative real time-PCR

Total RNA was isolated from sham-operated and the infarcted myocardium at 72 h after surgery. The expression of mRNA for interleukin-6 (*Il6*), *Il1β*, *Il10*, monocyte chemotactic protein-1 (*Mcp1*), matrix metalloproteinase-9 (*Mmp9*), macrophage migration inhibitory factor (*Mif*) were determined by quantitative real-time PCR (qPCR) and normalized by the housekeeping gene *18s*, as previously described [11]. The primer sequences are listed in Table 1

**Table 1:** Primer sequences for real-time qPCR.

	Forward primer	Reverse primer
*18s*	TTGACGGAAGGGCACCACCAG	GCACCACCACCCACGGAATCG
*Il6*	GCTACCAAACTGGATATAATCAGGA	CCAGGTAGCTATGGTACTCCAGAA
*Il1β*	AGGAGAACCAAGCAACGACA	GCTTGGGATCCACACTCTCC
*Mmp9*	CACCTTCACCCGCGTGTAC	TGCTCCGCGACACCAAA
*Mcp1*	CTGCATCTGCCCTAAGGTCT	AGTGCTTGAGGTGGTTGTGG
*Mif*	CCGGACCGGGTCTACATCAA	GGACTCAAGCGAAGGTGGAA
*Il10*	ACCTGGTAGAAGTGATGCCC	GGAGAAATCGATGACAGCGCC

### Hematological Examination

Animals were anesthetized using 2% isoflurane, 100 μl of blood was collected from the inferior vena cava in mice that underwent sham operation, I/R or MI 72 h after surgery. Heparin was used as an anticoagulant during the blood collection process. Hematological analysis was performed using Mindray auto hematology analyzer (BC-5300V) following the manufacturer’s instruction.

### Plasma analysis

Collected blood was centrifuged, and aliquots of plasma were stored at −80°C until analyzed. The plasma levels of IL-1β and MMP-9 were determined by enzyme-linked immunosorbent assay (ELISA) using EIAaB kits (E0563m and E00553m) according to the manufacturers’ procedures.

### Fluorescence Activated Cell Sorting (FACS)

Peripheral blood collected with heparin as the anticoagulant 72 h after surgery was used for FACS. Briefly, whole blood was layered on to the top of Ficoll-Paque and centrifuged at 500 *g* for 25 min at room temperature. The mononuclear cell layer was carefully collected, as previously described [19]. Collected mononuclear cells were resuspended in red blood cell (RBC) lysis buffer (BD Biosciences) and then washed with PBS. The cell number from cell suspensions of each sample was measured. The cell suspension from all samples were adjusted with PBS for the concentration of 2 × 10^6^. CD16/32 (1:200, 101302, Biolegend) antibody was used to blocked. FITC anti-CD45 (1:200, 103108, Biolegend), PE/Cy7 anti-CD11c (1:200, 117318, Biolegend), APC anti-CD115 (1:200, 135510, Biolegend), PerCP/Cy5.5 anti-CD11b (1:300, 101228, Biolegend), APC/Cy7 anti-mouse F4/80 (1:300, 123118, Biolegend) and PE-conjugated anti-Ly6-C (1:200, 128008, Biolegend) were used to identify Ly-6C^high^ monocytes in FACS analysis. Cells from each sample were incubated with the appropriate antibodies for 30 min at 4°C to label the cells and then analysed via FACS. All FACS analysis was performed on BD LSRFortessa™ X-20 and 300,000 total events per sample were collected. Cell analysis was performed using Flowjo10.0 (TreeStar). Cells were gated first based on size and granularity, using FSC-A forward scatter-area vs. SSC-A side scatter-area, to eliminate red blood cell and impurities. CD45 and CD11c were applied for determination of leukocyte population. This was followed by gating of CD115 positive cells and CD11b high to gate for monocytes only. The population of monocytes was identified as CD115+ CD11b+ and Ly-6C^high^ monocyte were identified as (F4/80− and Ly-6C+). Quantification of cell populations were expressed as cell numbers normalized by corresponding blood sample volume.

### Gelatin zymography

Abundant and activity of MMP-9 in the sham heart and the infarct myocardium were assessed at day-3 (72h) post-surgery. Total proteins were extracted and 100 μg of total protein lysate was separated by SDS gelatin gel. The gel was washed in 0.25% Triton X-100 for 30 min to remove excess SDS and then incubated 48 h at 37°C in incubation buffer (50 mM Tris pH 7.4, 10 mM CaCl_2_, 1% Triton X-100, 1 μM ZnCl_2_). The gel was stained in 0.1% Coomassie blue for at least 1 h and then de-stained (7% acetic acid, 20% methanol) for 2–4 h. The densitometry levels were quantified using ImageJ analysis, as described previously [20].

### Determination of tension-to-rupture (TTR)

The mechanical tensile strength of the LV myocardium was determined at day-3 after surgery, the time point is occurrence of peak cardiac rupture started [7]. The method has been described previously in details [7]. Briefly, fresh LV transverse sections (1-mm thickness) from the apex to the base of the LV were collected from each heart. LV rings containing infarct and/or non-infarct segments from MI and I/R model or only non-infarct segment from the sham heart were mounted on a stainless steel wire that was connected to a pre-calibrated force transducer (MLTF050/ST, ADInstruments, USA). The tension required to broken/rupture the LV ring was recorded by the PowerLab data acquisition system (ADInstruments, USA). The peak tension was taken as TTR for each ring and expressed as milli-newton (mN).

### Immunohistochemistry

LVs were harvested after 3, 5, 7 and 10 days of surgery. Fresh frozen LV sections at 5 μm thickness were used for immunofluorescent staining. For pan-leukocyte staining, LV sections were incubated with rat anti-mouse CD45 primary antibody (1:200 dilution, AB23910, Abcam). Subsequently, the sections were treated with secondary antibodies (Alexa Fluor® 546 goat anti-rat IgG, 1:500 dilution, red florescence for CD45, A-11081, Invitrogen). For type 2 and non-type 2 macrophages staining, LV sections were incubated with primary antibody mixture, including rat anti-mouse CD68 (1:200 dilution, ab53444, Abcam) and rabbit anti-mouse CD206 (1:50 dilution, 24595, Cell Signaling Technology). Then, the sections were treated with a mixture of secondary antibodies, comprising Alexa Fluor® 546 goat anti-rat IgG (1:200 dilution, red florescence for CD68, A-11081, Invitrogen) and Alexa Fluor® 488 goat anti-rabbit IgG (1:200 dilution, green florescence for CD206, AB150077, Abcam). Nuclei were stained by 4′,6-diamidino-2-phenylindole dihydrochloride (DAPI, 1:1000, 10236276001, Roche). Co-localization of red and green fluorescent colors turned to orange or yellow color identifying type 2 macrophage. Non-type 2 macrophages were identified as cells that exhibited only red fluorescence (CD68 positive) without green fluorescence (CD206 negative). For endothelial cells staining, LV sections were incubated with Alexa Fluor® 568 isolectin GS-IB4 conjugate (1:10 dilution, orange fluorescence, I21412, Invitrogen) overnight to stain endothelial cells. After PBS wash, sections were incubated with WGA-FITC solution (1:50 dilution, green fluorescence, FL1021, Vector Lab) to stain myocyte membrane for 2 h [10]. LV sections were covered with coverslips and visualized using a Leica DM6000B fluorescence microscope. Eight to ten images were obtained from each heart and assessed using Image-Pro Plus software in a blind fashion. For type 2 and non-type 2 macrophages, the number of each type of cells was counted and expressed as a percentage relative to the total cell (nuclei) count. For endothelial cells, the density was determined by calculating the number of endothelial cells per mm^2^.

### Detection of cardiomyocyte apoptosis

At 72 h after surgery, apoptotic cells in fresh frozen LV sections were estimated using In Situ Cell Death Detection Kit (12156792910, Roche Diagnostics), according to the manufacturer’s instructions. Fragmented nuclei were stained with red fluorescence. Nuclei were counterstained use DAPI, blue fluorescence, and the co-localization of red and blue fluorescence (pink colour) indicated apoptotic cardiomyocytes. Images of 9–11 visual fields from ischemic zone of each heart were acquired with Leica TCS SP8 confocal laser scanning microscope. The percentage of TUNEL-positive stained nuclei among the total number of nuclei were calculated as an apoptotic index [21].

### Echocardiography

Mice were anaesthetized with isoflurane at 4% for induction and 1.5–2% for maintenance. Transthoracic echocardiography was performed using Vevo 3100 ultrasound system equipped with 40 MHz linear-array transducer (VisualSonic Toronto, ON, Canada) at baseline, 1 and 4 weeks after surgery to determine the degree of LV remodeling and dysfunction after MI or I/R injury. Continual ECG monitoring was obtained via limb electrodes. Cardiac images of standard parasternal long-axis and short-axis were acquired. Images were analyzed by a single investigator in a blinded fashion using the Vevo 3100 analysis software (version 2.1.0). Heart rate (HR), LV end-systolic and diastolic diameters (LVESD, LVEDD), anterior and posterior wall thickness at the systole and diastole (AWs, AWd, PWs, PWd), fractional shortening [FS% = (LVEDD − LVESD)/ LVEDD × 100%], LV mass {LV mass = [(LVEDD + Awd + Pwd)^3 ^− LVEDD^3^] × 1.055} and LV mass index (LVMI = LV mass/ body weight) were calculated from the short-axis images. LV end-systolic and diastolic areas (LVESA, LVEDA), LV end-systolic and diastolic volumes (LVESV, LVEDV), stroke volume (SV = LVEDV − LVESV), cardiac output (CO = SV × HR) and ejection fraction (EF% = SV/ LVEDV × 100%) were calculated from the long-axis images.

### Histology

Hearts were harvested at 5, 7 and 10 days post-surgery and at the end of the study (4 weeks) and then fixed in 10% formalin. LV sections, sliced at a thickness of 5 μm, were obtained from regions close to the equator, including both the infarct and non-infarct segments. These sections were stained with hematoxylin-eosin (HE, for total cell density by counting nuclei, and coagulative necrotic myocardium) or 0.1% Sirius red (for collagen content). Microscopic images were captured at 20 × magnification from Sirius red stained sections to analyze fibrosis and at 10 × magnification to measure infarct wall thickness. For HE stained, LV sections were captured at 40 × magnification to quantify total cell density and at 10 × magnification to measure the size of coagulative necrotic myocardium. These images covered the entire infarct region and 6–8 sections were analyzed per heart. Measurements of collagen content, total cell density, and size of residual coagulative necrotic myocardium were performed using Image-Pro Plus 6.0 software (Media Cybernetics, Inc, USA). The total cell density was reported as the average number of cells per mm^2^. The coagulative necrotic area was expressed as a percentage of the entire infarct region [19]. The collagen positive stained area was calculated and expressed as a percentage within the infarct area, as described previously [22].

### Statistical analysis

Results for continuous variables were expressed as mean±SEM. The Kolmogorov–Smirnov test indicated that the data had a normal distribution. GraphPad Prism software (version 7.0) was used for analysis. One-way and two-way ANOVA were used for overall significance and followed by Tukey’s multiple comparison post-*hoc* test. Correlation analysis between LV mass and LV weight was performed using Pearson’s correlation analysis. For incidence of rupture, Fisher exact tests were performed as appropriate. *P < 0.05* was considered statistically significant.

## Results

### Early and delayed reperfusion following myocardial ischemia prevents fatal cardiac rupture

**Fig 1:**
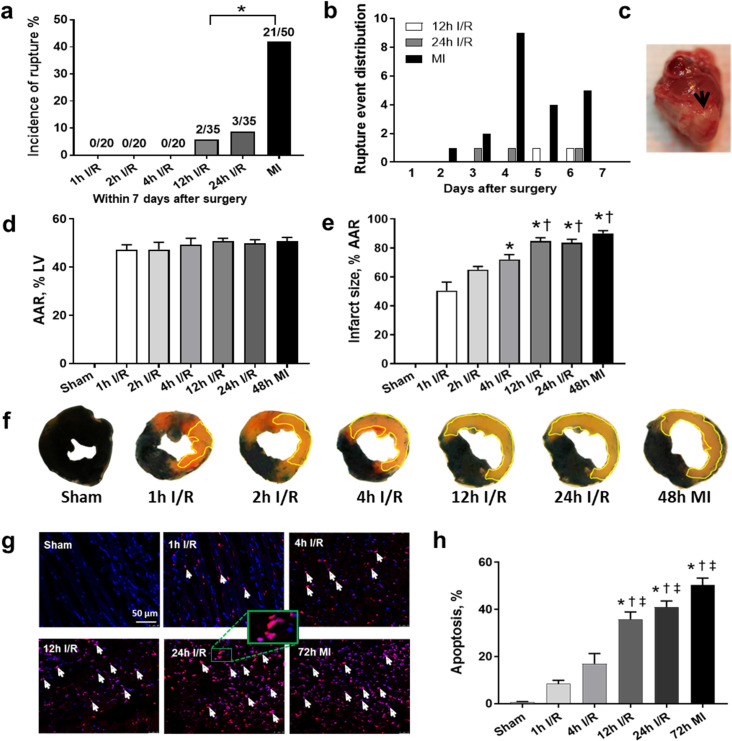
Incidence of cardiac rupture, infarct size and myocyte apoptosis in early and delayed reperfusion and MI groups. (a) Cumulative incidence of cardiac rupture in different groups within 7 days after surgery. (b) Time distribution of cardiac rupture occurrence. (c) Representative images of cardiac rupture. (d) Quantitative data to show percentages of the area at risk (AAR) to total LV area in sham, I/R and MI groups at day 3 after surgery, n = 6–9 per group. (e) Quantitative data to show percentages of infarct size to AAR in sham, I/R and MI groups at day 3 after surgery, n = 6–9 per group, *P < 0.05 vs. 1h I/R. †*P < 0.05* vs. 2h I/R. (f) Representative LV section stained by Evans blue and TTC to depict the size of infarcted myocardium. The area where the myocardium turned pale is the infarct area (yellow line). Red and pale color areas indicate AAR and the nonischemic myocardium is blue. (g) Representative images of TUNEL staining at day 3 after surgery. Overlapping (pink color) of blue-stained DAPI and TUNEL-positive nuclei (red color) indicate apoptotic cells. The inset shows the enlarged image of typical TUNEL-positive stained nuclei. Scale bar = 50 μm. (h) Quantitative data to show percentages of TUNEL-positive stained nuclei among the total number of nuclei cells, n = 5 per group, *P < 0.05 vs. sham. †*P < 0.05* vs. 1h I/R. ‡ *P < 0.05* vs. 4h I/R.

In total, 20 mice each from 1h, 2h and 4h I/R groups, 35 mice each from 12h and 24h I/R groups and 50 mice with MI survived after surgery were monitored for up to 10 days. There was no cardiac rupture or heart failure death in 1h, 2h and 4h I/R groups. We observed 0.4-fold MI mice died of cardiac rupture (Fig 1a). While mice subjected to 12 h or 24 h ischemia following reperfusion had a significantly low incidence of cardiac rupture (5.7% or 8.6%, Fig 1a). Autopsy on dead mice showed that cardiac rupture and heart failure were the major cause of death during the first 7 days post-surgery. Cardiac rupture occurred during days 2–7, most frequently between 4–6 days (Fig 1b). The size of rupture varied from a fine perforation to a 2–3 mm slit (Fig 1c). The heart with LV free wall rupture commonly displayed regional dilatation and intra-myocardial hemorrhage. Heart failure death was only found in mice subjected to 12h I/R (11.4%, 4/35), 24h I/R (11.4%, 4/35) and MI (12%, 6/50). The 2h I/R group was added only for rupture incidence and infarct size analysis.

### Prolonged ischemia resulted in larger infarct size and more myocyte apoptosis

To determine the influence of ischemic duration in the extent of myocardial injury, infarct size was assessed 48h after surgery. At the ‘same’ ligation level indicated by a similar area at risk (AAR, Fig 1d), prolongation of ischemic time increased infarct size gradually (Fig 1e,f). Compared to mice with 1h I/R (51%), infarct size in 2h and 4h I/R group marginally increased Unexpectedly, infarct size in 12h I/R, 24h I/R and 48h MI groups were comparable (84%−90%) albeit there was a significant increase compared to 1h and 2h I/R groups (*P < 0.05*). Further, TUNEL staining performed at 3 days after ischemia (the peak timing of inflammatory response) revealed a stepwise increase of myocyte apoptosis along with extension of ischemic duration despite of reperfusion (Fig 1g,h). Compared to sham group, myocyte apoptosis increased by 12–25 folds in 1h and 4h I/R group, while had a dramatic augmentation (55–78 folds) in 12h and 24h I/R and MI groups (Fig 1h).

### Prolonged ischemia enhanced systemic inflammatory response despite of reperfusion

**Fig 2:**
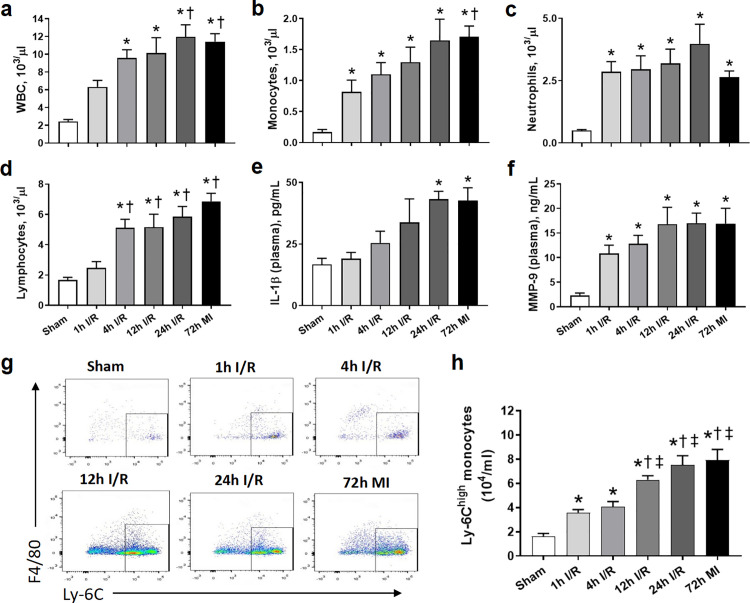
Hematological profile, plasma levels of inflammatory cytokines, Ly-6C^high^ monocyte populations from peripheral blood in different groups at 72 h after surgery. (a) Counts of white blood cells (WBC) in peripheral blood. n = 5-7 per group. (b) Counts of monocytes in peripheral blood. n = 5-7 per group. (c) Counts of neutrophils in peripheral blood. n = 5-7 per group. (d) Counts of lymphocytes in peripheral blood. n = 5-7 per group. (e) Plasma levels of IL-1β. n = 5-8 per group. (f) Plasma levels of MMP-9. n = 5-8 per group. (g) Representative scatter plots of Ly-6C^high^ monocytes from peripheral blood. (h) Quantification of Ly-6C^high^ monocytes from peripheral blood. n = 7-13 per group. *P < 0.05 vs. sham. †*P < 0.05* vs. 1h I/R. ‡ *P < 0.05* vs. 4h I/R.

The inflammatory response reaches its peak on day 3 after MI [23]. Therefore, systemic inflammation was assessed by routine blood test for different groups at 72 h port-surgery. Compared to the sham value, the change of WBC and monocyte counts had a similar trend with 2–4 folds elevation in 1h I/R group and a further increased in other ischemic groups (Fig 2a,b). Neutrophil counts had a 4–7 folds increase in all ischemic groups and the values were comparable among ischemic groups (Fig 2c). Lymphocyte count was only slightly increased in 1h I/R group, but raised by 2–3 folds in other ischemic groups (Fig 2d). Notably, in most case, there was no statistical different in these cell counts among 4h, 12h and 24h I/R and MI groups and the highest values were observed in 24h I/R and MI groups.

Further, we also measured plasma levels of inflammatory cytokines by ELISA and Ly-6C^high^ monocyte populations by FACS from peripheral blood at the same time. Circulating IL-1β levels did not change in 1h I/R group and only slightly elevated in 4h I/R group, but there was a 1–1.6 folds increase in 12h and 24h I/R and MI groups, respectively (Fig 2e). Unlike IL-1β, MMP-9 levels had a comparable 4−6 folds increase in all ischemic groups versus the sham level (Fig 2f). Ly-6C^high^ monocyte populations was similarly raised by 1.3 folds in both 1h and 4h I/R groups, and there was a further 0.5–0.9 fold increase in 12h and 24h I/R and MI groups (Fig 2g,h).

### Prolonged ischemia augmented regional inflammatory response

**Fig 3:**
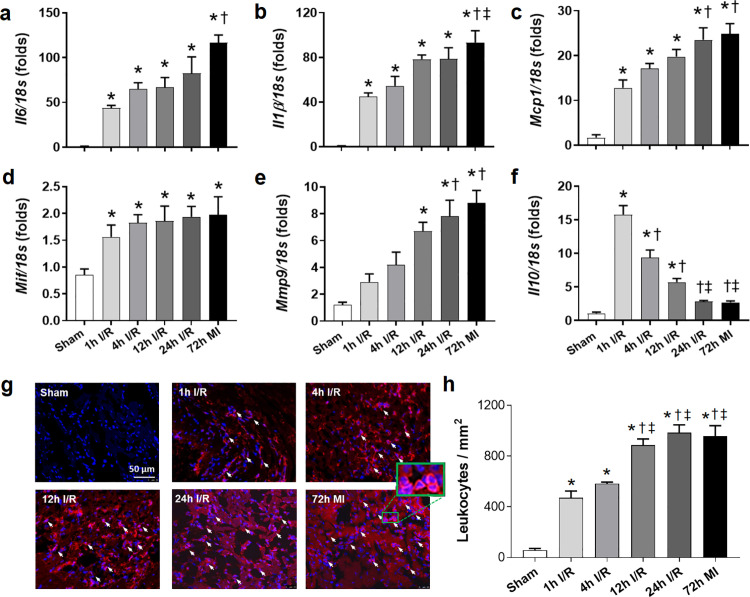
Gene expression of inflammatory cytokines and regional leukocyte infiltration in different groups at 72 h after surgery. (a) mRNA levels of interleukin-6 (*Il6*) in the infarct tissue. n = 5-6 per group. (**b**) mRNA levels of *Il1β* in the infarct tissue. n = 5-6 per group. (**c**) mRNA levels of monocyte chemoattractant protein-1 (*Mcp1*) in the infarct tissue. n = 5-6 per group. (**d**) mRNA levels of macrophage migration inhibitory factor (*Mif*) in the infarct tissue. n = 5-6 per group. (**e**) mRNA levels of matrix metalloproteinase-9 (*Mmp9*) in the infarct tissue. n = 5-6 per group. (**f**) mRNA levels of *Il10* in the infarct tissue. n = 5-6 per group. Data are expressed as fold change compared with sham group. (g) Representative images of CD45 positive stained cells in the infarct region. The pink colour indicates overlap of CD45 staining (red) with DAPI (blue) staining for nuclei. The inset shows the enlarged image of CD45 positive stained cells. Scale bar = 50 μm. (h) Quantification of leukocytes (CD45 positive cells) number. Data were expressed as the number of CD45 positive cells per mm^2^. n = 4 per group. *P < 0.05 vs. sham. †*P < 0.05* vs. 1h I/R. ‡ *P < 0.05* vs. 4h I/R.

To evaluate the local myocardial inflammatory response prior to the peak of cardiac rupture (4 days after ischemia, Fig 1b), we tested mRNA expression of pro- and anti-inflammatory cytokines and inflammatory cells infiltration in the infarcted LV tissues 72 h after surgery. mRNA levels of *Il6*, *Il1β* and *Mcp1* all had dozens of times increase over their sham levels after ischemic insult with the highest level in MI group (Fig 3a–c). A comparable 0.8-1.3-fold elevation was observed in *Mif* expression in all ischemic groups (Fig 3d). *Mmp9* expression displayed a progressive increase from 1.4 to 2.5 folds in 1h and 4h I/R group to 4.6–6.3 folds in 12h and 24h I/R and MI groups and the highest level was detected in MI group (Fig 3e). Contrarily, anti-inflammatory cytokine, *Il10*, had a sharp rise (~14 folds) in 1h I/R group and with a stepwise decrease to 7.5, 4.2 and 1.5 folds in 4h, 12h and 24h I/R and MI groups from the sham level, respectively (Fig 3f).

Corresponding to elevated pro-inflammatory cytokine expression, we also observed a progressively increase in inflammatory cell infiltration in the ischemic/infarcted myocardium. Leukocyte density increased in 1h and 4h I/R group by 7–9 folds and it was further raised to a similar level of 14–16 folds in delayed reperfusion (12h and 24h I/R) and MI groups (Fig 3g,h). These results demonstrated a beneficial anti-inflammatory effect from an early reperfusion following prolonged ischemia.

### Prolonged ischemia weakened the tensile strength of myocardium, which was corresponding to MMP-9 activity

**Fig 4:**
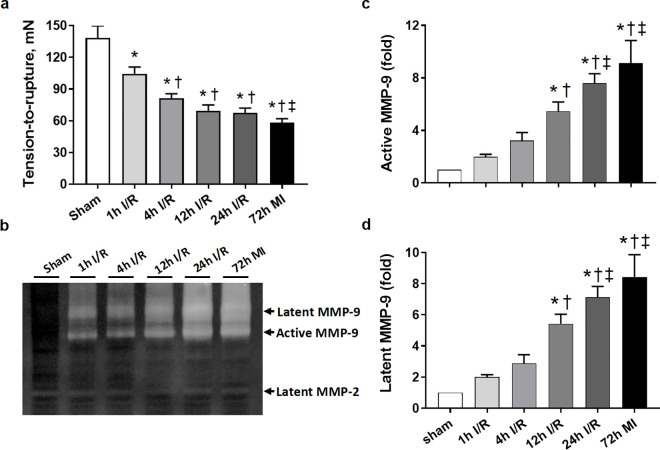
The tensile strength of myocardium and matrix metalloproteinase (MMP-9 and −2) activity in different groups at 72 h after surgery. (a) Changes of tensile strength of myocardium. n = 13 for sham group, n = 25-43 per ischemic group. (b) Representative images of gelatine zymography demonstrating changes of active and latent MMP-9 and MMP-2. (c) Quantitative analyses of active MMP-9 by zymography in the infarct tissue. n = 3-4 per group. (d) Quantitative analyses of latent MMP-9 by zymography in the infarct tissue. n = 3-4 per group. Data are expressed as fold change compared with sham group. *P < 0.05 vs. sham. †*P < 0.05* vs. 1h I/R. ‡ *P < 0.05* vs. 4h I/R.

The tensile strength of LV rings and activation of MMP-9 were determined at the peak of inflammatory response on day-3 after surgery. TTR of the non-infarcted LV rings from all ischemic models were similar as it from the sham hearts. Compared to TTR from the sham heart, there was a stepwise reduction in TTR (from 0.3 to 0.6 fold) from hearts subjected to ischemia followed by early and delayed reperfusion and 72h MI (all *P < *0.05, Fig 4a). These results indicate better preserved tensile strength after reperfusion compared with MI mice and particularly in early reperfusion hearts, which is in keeping with a lower risk of cardiac rupture in these hearts.

Activation of MMPs, which degrading extracellular matrix (ECM), plays a critical role in the pathogenesis of cardiac rupture and remodeling post-MI [24]. Compared to the sham value, levels of active MMP-9 elevated by 1, 2.2, 4.5, 6.6 and 8.1 folds in 1h, 4h, 12h and 24h I/R and MI groups, respectively, the highest level was detected in MI hearts (Fig 4b,c). Similarly, latent MMP-9 levels also showed a similar trend (Fig 4b,d). These results suggest that reperfusion mitigated activation of latent and active MMP-9 in the infarcted tissue. There was no significant change in MMP-2 activity at this time point (Fig 4b).

### Early reperfusion attenuated adverse cardiac remodeling and dysfunction

**Fig 5:**
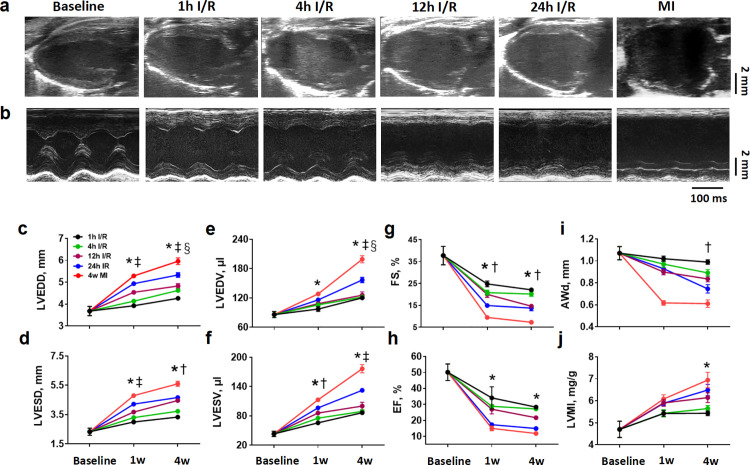
Echocardiographic analysis for influence of different ischemic protocols in cardiac remodeling and dysfunction. (a) Representative B-mode views of long-axis at the left ventricular end-diastole (LVED) from different groups at 4 weeks after surgery. (b) Representative M-mode views of short-axis at the LVED from different groups at 4 weeks after surgery. Horizontal scale bar = 100 ms and vertical scale bar = 2 mm. (c) Changes in LV end-diastolic diameter (LVEDD) at baseline, 1 and 4 weeks after surgery. (d) Changes in LV end-systolic diameter (LVESD) at baseline, 1 and 4 weeks after surgery. (e) Changes in LV end-diastolic volume (LVEDV) at baseline, 1 and 4 weeks after surgery. (f) Changes in LV end-systolic volume (LVESV) at baseline, 1 and 4 weeks after surgery. (g) Changes in fractional shortening (FS) at baseline, 1 and 4 weeks after surgery. (h) Changes in ejection fraction (EF) at baseline, 1 and 4 weeks after surgery. (i) Changes in anterior wall thickness at the diastole (Awd) at baseline, 1 and 4 weeks after surgery. (j) Changes in LV mass index (LVMI) at baseline, 1 and 4 weeks after surgery. n = 8–12 per group. *P < 0.05, 24h IR and MI vs. baseline at same time point. †*P < 0.05*, 24h IR and MI vs. 1h and 4h IR at same time point. ‡ *P < 0.05* 24h IR, MI vs. 1h, 4h IR and 12h IR at same time point. § *P < 0.05* 1w vs. 4w time point in 24h IR and MI groups.

We assessed LV structure and function by echocardiography pre-operatively and at weeks 1 and 4 post-surgery. Echocardiographic data from long-axis and short axis are presented in S1 and S2 Tables. Mice with relative normal function and less chamber dilation, an indication of small infarct, were excluded from data analysis. Representative echo images are displayed in Fig 5a–b. Heart rates among different groups were similar at these time points. As shown in S1 and S2 Tables and Fig 5c–f, a LV chamber dilatation (LVEDD, LVESD, LVEDA, LVESA, LVEDV, LVESV) was detected at 1 week and becoming prominent 4 weeks post-surgery compared to the baseline level. The chamber dilatation at week-1 was more evident in delayed reperfusion (12h and 24h I/R) and MI groups, and the most severe dilatation was detected in 24h I/R and MI groups by week-4. Along with LV chamber dilatation, contractile function was declined. Both FS and EF had a significant fall (FS from 0.5 to 3.1 folds; EF from 0.5 to 2.3 folds) at week-1 in all ischemic groups and the largest amplitude was observed in 24h I/R and MI groups and this trend continued to week-4 (Fig 5g–h). SV and CO are another kind of functional parameters. At 1 week after surgery, significant decrease in SV only detected in delayed reperfusion (12h and 24h I/R) and MI groups, while CO decrease was detected in all ischemic groups. At week-4, SV and CO remained virtually unchanged or slightly reversed (S2 Table). LV wall thickness was largely unchanged, a significant thinning was detected in 24h I/R and MI groups (S1 Table) and MI hearts had the most severe wall thinning at week-4 (Fig 5i). Prominent increase of LVMI was noticed in MI group as early as week-1 post surgery and a further increase at week-4 was found in delayed reperfusion (12h and 24h I/R) and MI groups. Notably, LV mass correlated positively with LV weight in early reperfusion (1h and 4h I/R) groups (S1a Fig). However, this correlation was lost in delayed reperfusion (12h and 24h I/R) and MI groups (S1b,c Fig).

### Prolonged ischemia led to a higher incidence of heart failure and more severe cardiac fibrosis

**Fig 6:**
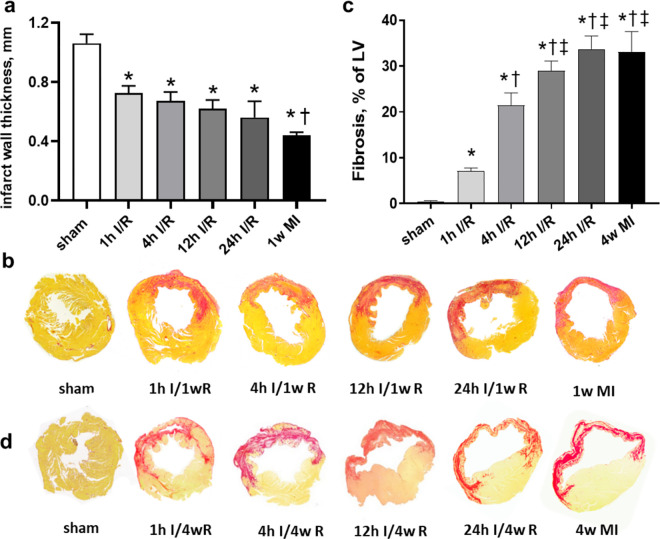
Change of infarct wall thickness at 1 week after surgery and fibrosis at 4 weeks after surgery in different groups. (a) Quantification of infarct wall thickness. n = 4-5 per group. (b) Representative LV cross-sections stained with Sirius red at 1 week after surgery. (c) Quantification of fibrosis. Data are expressed as a percentage within the infarct area. n = 8-12 per group. (d) Representative LV cross-sections stained with Sirius red for scar tissue (fibrosis) at 4 weeks after surgery. *P < 0.05 vs. sham. †*P < 0.05* vs. 1h I/R. ‡ *P < 0.05* vs. 4h I/R.

At the end of study, organs were harvested and morphological parameters were measured and presented in Table 2. Delayed reperfusion (12h and/or 24h I/R) and MI groups had a significantly increased LV weight evidenced by a higher LVW/BW and LVW/TL ratio compared to the sham value, this was not the case in early reperfusion (1h and 4h I/R) groups. A clear trend of increased lung weight was observed in association with extension of ischemic duration and a marked increase of lung weight was only detected in MI group. In addition, mice subjected to short-term ischemia (1h and 4h I/R) had not pleural effusion and atria thrombus detected. Whereas, incidences of pleural effusion and atria thrombus increased in 12h I/R, 24h I/R and MI groups (Table 2). Moreover, we also examined infarct wall thickness and the size of scar tissue histologically. Compared with sham group, infarct wall thickness progressively decreased in all ischemic groups with a thinnest LV wall in MI group at 1 week post-surgery (*P < 0.05,* Fig 6a). Fig 6b and d showed the representative images of LV sections by Sirius red staining at 1 week or 4 weeks post-surgery. Either in I/R or MI hearts, the red-stained area is coincided with the scar tissue. In short ischemia (1h and 4h I/R) groups, scar tissue was staggered with survived myocardium, yet it was transmural in prolonged I/R and MI hearts. Quantitative analysis showed a progressively increased collagen content with prolongation of ischemic duration, indicating a development of fibrosis (Fig 6c).

**Table 2:** Morphological parameters and incidence of pathological events in the different groups determined at the end of 4-week study.

Variables	Sham (n = 10)	1h I/R (n = 12)	4h I/R (n = 10)	12h I/R (n = 9)	24h I/R (n = 8)	4w MI (n = 9)
BW (g)	26 ± 1	28 ± 1	28 ± 1	28 ± 1	28 ± 1	28 ± 1
TL (mm)	18.1 ± 0.0	18.2 ± 0.1	18.3 ± 0.1	18.2 ± 0.1	18.2 ± 0.1	18.3 ± 0.1
LVW (mg)	86.6 ± 1.7	100.8 ± 2.9	105.9 ± 4.1	124.3 ± 7.1^*^	140.1 ± 6.8^*†‡^	166.3 ± 10.3^#^
Lung W (mg)	149.7 ± 6.0	165.1 ± 5.2	167.4 ± 8.2	170.0 ± 5.8	181.9 ± 4.6	198.7 ± 10.0^*†^
LVW/BW (mg/g)	3.4 ± 0.1	3.6 ± 0.1	3.7 ± 0.2	4.5 ± 0.3	5.0 ± 0.2*	6.0 ± 0.3^*†‡^^ψ^
Lung W/BW (mg/g)	5.8 ± 0.2	5.9 ± 0.2	6.0 ± 0.4	6.1 ± 0.2	6.5 ± 0.2	7.2 ± 0.3
LVW/TL (mg/mm)	4.8 ± 0.1	5.5 ± 0.1	5.8 ± 0.2	6.8 ± 0.4^*^	7.7 ± 0.4^*†‡^	9.1 ± 0.6^#^
Lung W/TL (mg/mm)	8.2 ± 0.3	9.1 ± 0.2	9.2 ± 0.4	9.4 ± 0.3	10.0 ± 0.2	10.9 ± 0.6^*†^
PE (%)	0	0	0	57.1^*†‡^	44.4^*†‡^	40.0^*†‡^
Ath (%)	0	0	0	21.4^*†‡^	22.2^*†‡^	33.3^*†‡^

Values are mean ± SEM. 1h I/R, 4h I/R, 12h I/R, 24h I/R, mice subjected to 1h, 4h, 12h or 24h ischemia followed by 4 weeks reperfusion; MI, myocardial infarction; BW, body weight; TL, tibial length; LVW, left ventricle weight; PE, pleural effusion; Ath, Atria thrombus.

* *P < 0.05* vs. sham. ^†^*P < 0.05* vs. 1h I/R group. ^‡^*P <* *0.05* vs. 4h I/R group. ^ψ^*P < 0.05* vs. 12h I/R group. #*P < 0.05* vs. all other groups.

### Accelerated healing as a mechanism of delayed reperfusion to prevent cardiac rupture

**Fig 7:**
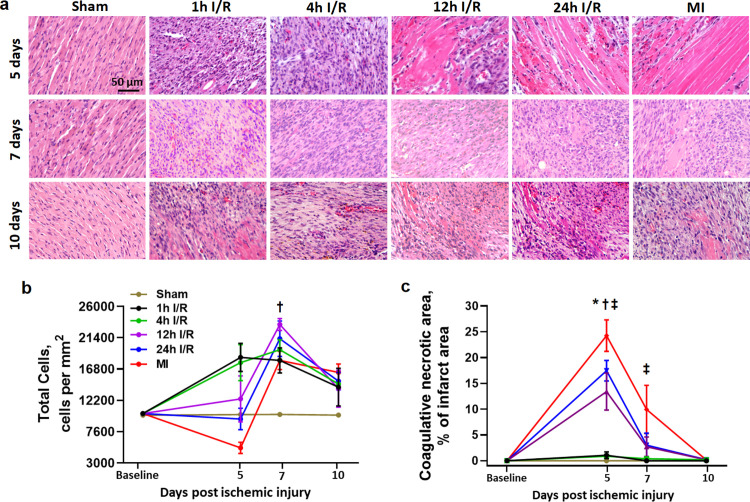
The changes of the total cell number and the size of coagulative necrotic myocardium in the infarct region from different groups at 5, 7 and 10 days after surgery. (a) Representative images of HE stained left ventricular (LV) sections. Scale bar = 50 μm. (b) Quantification of total cell counting. Data were expressed as number of nuclei per mm^2^. (c) Changes in the size of the coagulative necrotic myocardium. Data were expressed as a percentage of the entire infarct region. n = 4 per group. *P < 0.05 12h, 24h IR and MI vs. 1h and 4h IR at same time point. †*P < 0.05* 12h and 24h IR groups vs. sham at same time point. ‡ *P < 0.05* MI group vs. sham at same time point.

To assess the potential underlying mechanisms of delayed reperfusion in preventing fatal cardiac rupture following ischemia, we measured the total number of cells, type 2 and non-type 2 macrophages, endothelial cells and the size of coagulative necrotic myocardium in the infarct zone at 5, 7 and 10 days post-surgery. These time points were selected as they fall within the time window of cardiac rupture occurrence [8]. The number of total cells exhibited an initial increase within the first 7 days in the early reperfusion (1h, 4h I/R) groups, followed by a gradual decrease thereafter (Fig 7a,b). In the delayed reperfusion (12h, 24h I/R) groups, there was no significant change in total cell density on day 5 after surgery, whereas, a significant elevation was observed between days 5 and 7 (Fig 7a,b). The total cell density exhibited a decreasing trend at 5 days after MI (due to the large coagulative necrotic area) followed by a substantial 2.4-fold increase at 7 days post-MI. On the 10th day after surgery, the delayed reperfusion groups demonstrated a significant decline in total cell density. However, there was no change in total cell density observed in the MI groups (Fig 7a,b). Coagulative necrosis is a vital indicator for evaluating myocardial healing. The size of coagulative necrotic myocardium in the early reperfusion groups exhibited a marginal increase at 5 days post-ischemia, subsequently returning to sham levels between days 5 and 7 (Fig 7a,c). In contrast, the delayed reperfusion groups (12h and 24h I/R) and MI groups demonstrated significantly larger necrotic areas at 5 days after ischemia (Fig 7a,c). On the day-7, the areas of coagulative necrotic area markedly shrunk in both the delayed reperfusion and MI groups with a relatively large size in MI group (Fig 7a,c). By day-10, the coagulative necrotic myocardium virtually disappeared across all surgery groups (Fig 7a,c).

**Fig 8:**
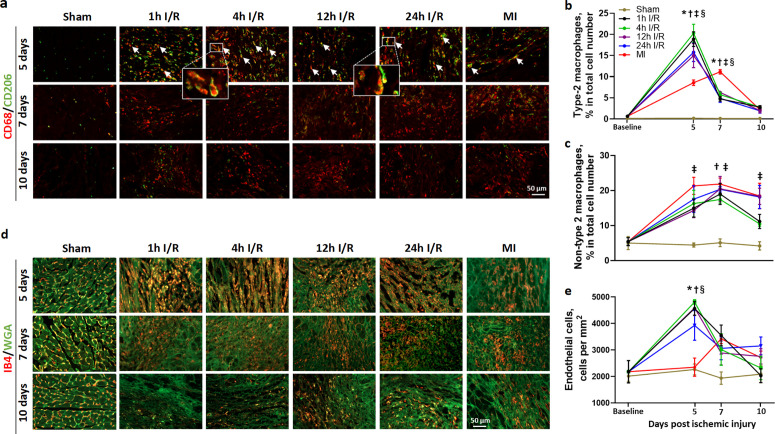
Densities of type 2 and non-type 2 macrophages and endothelial cells in different groups at 5, 7, 10 days after surgery. (a) Representative images of type 2 macrophages (CD68 and CD206 double-immunofluorescent staining) in the infarct region. The yellow or orange colour (arrows) indicates overlap of CD68 (red) and CD206 (green) positive staining. Two amplified images (inset) show a typical overlap staining. Scale bar = 50 μm. (b) Quantification of the type 2 macrophages. Data were expressed as a percentage of the number of total cells. (c) Quantification of the non-type 2 macrophages (most were type 1 macrophages). Data were expressed as a percentage of the number of total cells. (d) Representative images showing endothelial cells density in the infarct region. IB4 (red) staining for endothelial cells and WGA (green) staining for myocardial cells. Scale bar = 50 μm. (e) Quantification of the endothelial cells numbers. Data were expressed as the number of the endothelial cells per mm^2^. n = 3-4 per group. *P < 0.05 1h and 4h IR vs. sham at same time point. †*P < 0.05,* 12h and 24h IR vs. sham at same time point. ‡ *P < 0.05* MI vs. sham at same time point. § *P < 0.05* MI vs. 1h, 4h, 12h, 24h IR groups at the same time point.

Furthermore, we assessed the dynamic change of specific types of cells that are closely associated with cardiac repair. Type 2 macrophages also known as anti-inflammatory or pro-reparative macrophages, play a significant role in the quality and outcome of cardiac healing [25]. By day-5, the levels of type 2 macrophages (both CD68 and CD206 positive stained cells) were significantly increased in both I/R and MI groups with the lowest level in MI hearts. There was a sharp decline on day 7 in all I/R groups, by contrast, a further increase on day-7 in MI group was observed when compared to all I/R groups (Fig 8a,b). On day-10, the levels of type 2 macrophages decreased in all ischemic groups (Fig 8a–b). To further elucidate the phenotype shift between type 1 and type 2 macrophages during the critical early healing period, we also analyzed the non-type 2 macrophages, which are predominantly composed of type 1 macrophages, according to the classical polarization theory [26]. The number of non-type 2 macrophages augmented approximately 2–4 folds from the baseline level in all ischemia groups maintained a relatively high level from 5 to 7 days after surgery with the highest level detected in MI and delayed I/R group albeit there were no statistical differences among ischemia groups. By day-10, a decline is noted in the early reperfusion group, whereas no significant changes were observed in delayed reperfusion and MI groups (Fig 8c).

Endothelial cells are known to play an important role in angiogenesis during healing. Compared to sham hearts, there was a significant increase in the number of endothelial cells (IB4-positive stained cells) in all ischemic groups. The highest density of endothelial cells was observed at 5 days in all I/R groups, with the highest levels in the early reperfusion (1h and 4h I/R) groups. This density decreased to baseline levels by 10 days after ischemia in the early reperfusion groups (Fig 8d,e). In delayed I/R groups, the density of endothelial cells almost halved by day 7 from the peak of day 5 and maintained at the similar levels by 10 days after surgery (Fig 8d,e). In the MI group, the number of endothelial cells did not show a significant elevation on day 5 but increased by 0.5 fold on day 7 and subsequently, a declining trend was observed on day-10 post-MI. The density of endothelial cells was comparable among the delayed I/R and MI groups at 7–10 days post-ischemia (Fig 8d,e).

## Discussion

In this study, we examined the effects of different ischemic protocols with or without reperfusion on preventing cardiac rupture and mitigating adverse cardiac remodeling. We have made several major findings. *First*, either early or delayed reperfusion effectively prevented occurrence of fatal cardiac rupture, early reperfusion following 1–4 h ischemia completely diminished rupture occurrence. *Second,* early reperfusion was associated with alleviated acute inflammatory response compared with delayed reperfusion or ischemic insult without reperfusion. *Third*, early reperfusion significantly attenuated MMP-9 activities at 3 days post-ischemia thereby preserving tensile strength of the infarcted myocardium. *Fourth,* early reperfusion efficiently mitigated adverse cardiac remodeling post-ischemia. *Finally*, both early and delayed reperfusion promoted type 2 macrophage proliferation and angiogenesis at the 5^th^ day post-ischemia which facilitated the healing process.

Many clinical and experimental studies have recognized the critical role of ischemic duration in determining the extent of myocardial injury [27,28]. However, there is a paucity to elucidate the how ischemic duration influences myocardial injury and whether delayed reperfusion following prolonged ischemia is still able to bring benefits to prevent fatal outcomes. Using different I/R protocols, we found that 1 h ischemia in mice led to a 50% necrosis. When ischemic time was extended to 4 h, the infarct size reached approximately 70%. Further prolonging ischemia to 12–24 hours, i.e., delayed reperfusion, resulted in an even greater increase in infarct size, reaching over 80%, which was comparable with MI group. Synchronized increase of programmed cell death, myocyte apoptosis, was accompanied with necrosis development. Some studies have documented that when ischemic time extends 6 h, the myocardium would experience irreversible ischemic insult, i.e., necrosis phase [28]. Notably, although prolonged ischemia did led to more severe myocardial necrosis, we found that early reperfusion followed by 1 h and 4 h coronary artery occlusion completely diminished fatal cardiac rupture and precluded severe LV dilatation and dysfunction. Even delayed reperfusion, followed by 12 h and 24 h ischemia, largely prevented these adverse events. Impressively, delayed reperfusion followed by 12 h ischemia also substantially attenuated adverse cardiac remodeling. Considering this characteristic, the delayed reperfusion model may serve as a practical tool that can induce significant myocardial damage and cardiac remodeling while reducing significant loss of mice due to cardiac rupture death.

Following ischemia, injured cardiac cells trigger regional and systemic immune response and regional inflammatory mediators initiate and regulate healing process through degradation of ECM, phagocytosis, angiogenesis, collagen synthesis and scar formation [23]. In this study, we examined a range of systemic and regional inflammatory parameters and observed (1) a significant increase of circulating inflammatory cell numbers including WBC, neutrophils, monocytes and lymphocytes; (2) elevated circulating levels of IL-1β and MMP-9; (3) raised Ly-6C^high^ monocyte populations in peripheral blood; (4) surged regional leukocyte infiltration and (5) upregulated mRNA expression of inflammatory cytokines in the infarct tissue. Degree of all these changes augmented alone with extension of ischemic duration, which the maximal amplitude was detected in delayed reperfusion (12h, 24h I/R) and MI groups, indicating the most intense systemic and regional inflammatory response in these groups. Compared to delayed reperfusion, early reperfusion limited further progress of tissue necrosis, thereby associated with less extent of systemic and regional inflammatory response. It is conceivable that early reperfusion may also expedite the absorption of necrotic myocardium and consequently promote infarct healing. Our further mechanistic studies confirmed this hypothesis.

TTR is a useful surrogate to quantitatively measure mechanical strength of the myocardium [7]. Our results showed that decline of TTR was associated with extension of ischemia duration, the lowest TTR was observed in MI group. This phenomenon corresponded to the incidence of fatal cardiac rupture in mice with different ischemic interventions. ECM, composed of collagen, elastin, fibrillin, fibronectin network, is a multifunctional complex contributing to the structural and functional integrity of the heart [29]. Prolonged ischemia not only causes cardiomyocyte death but also ECM damage. Activation of MMPs, particularly MMP-9 mainly derived from infiltrated inflammatory cells, is the key driven force for degradation of ECM [8]. Indeed, we observed a stepwise increase of MMP-9 activity in murine ischemic myocardium at 72 h after attack which was coincident with extension of ischemia duration, degree of regional inflammatory response and most importantly incidence of cardiac rupture. Thus, activation of MMPs leading to degradation of ECM and consequent weakening of muscle tensile strength, is the central mechanism underlying the pathogenesis of rupture [24,30,31]. Noteworthily, substantial increase of MMP-9 activity in delayed (12h, 24h I/R) reperfusion groups, especially 24h I/R group, was not associated with a higher frequency of cardiac rupture like that in MI group, instead, occurrence of rupture was largely prevented. The possible explanation is that even delayed reperfusion is still able to rescue certain amount of ischemic myocardium and reduce formation of transmural infarction, thereby preventing fatal rupture occurrence. Along with such protective benefit, we also observed that even reperfusion delayed from 4 h up to 12 h after ischemic attack, which substantially prevented severe LV dilatation and function decline when compared to MI and 24h I/R groups. This finding highlights the importance of reperfusion therapy. The efficacy of delayed reperfusion is controversial [32–34], most clinical practices still pursue the “golden standard” 1-3 h time window of symptom-to-balloon” and the delayed reperfusion is not a routine practice [35,36]. However, pre-clinical animal studies have demonstrated convincing benefits from a delayed reperfusion [37–39] including findings from our current study. Taken together, even delayed reperfusion is still be able to rescue limited reversible myocytes, which confers protective benefits not just in infarct size limitation also in maintenance of LV wall strength [28].

The beneficial effects of delayed reperfusion in improving outcomes of myocardial infarction have been reported in many studies [40,41], however, there has been limited research exploring the underlying mechanisms behind these effects. The current study aimed to fill this gap by examining several indices related to myocardial healing within the acute phase. A strong correlation has been reported between wall thinning and decreased cell density in both rat and human hearts during the early stages of MI [42]. In the early reperfusion groups, the total cell density was maintained at a high level, which may reflect a “expedited” healing. In contrast, a very low total cell density observed at 5 days following permanent coronary artery occlusion may implicate a “delayed” healing. The size of coagulative necrosis also reflects healing process, the larger coagulative necrosis, the slower healing [43]. This was supported by the largest size of coagulative necrotic zone in MI hearts at both 5−7 days post-surgery but not in reperfusion groups. During the healing process, infiltrated macrophages undergo a phenotypic switch from a pro-inflammatory type 1 macrophage phenotype to a reparative type 2 macrophage phenotype [26,44]. We observed the density of both type 2 and non-type 2 macrophages (most were type 1 macrophages) increased with the most significant changes in type 2 macrophages at day-5 after ischemia, the critical early healing period. A clear trend was observed: the highest levels of type 2 macrophages and the lowest levels of non-type 2 macrophages were detected in the early reperfusion and 12h I/R groups. In contrast, the opposite pattern was seen in the MI and 24h I/R groups. These phenomena may imply a deferred and weakened angiogenesis during the critical early healing process (5 days post-ischemia) which corresponding to a larger coagulative necrotic area in the delayed 24h I/R and MI groups.

There are several limitations in our study. (1) we exclusively use of male mice. As estrogen has been shown to reduce inflammation, oxidative stress, apoptosis and infarct size, as well as to promote angiogenesis, which could significantly influence MI outcomes. Therefore, our findings may not be directly applicable to female mice. (2) Our study primarily focused on the inflammatory response (3 days post-ischemia) of the acute phase and the early repair phase (5–10 days post-ischemia). This design may limit a comprehensive understanding of the healing process influenced by reperfusion. (3) The reperfusion was only performed after 1, 4, 12, and 24 h ischemia. Further study may be required to overcome these limitations to gain better and comprehensive understanding in healing process influenced by reperfusion following prolonged ischemic insult.

In conclusion, findings from our study suggest that delayed reperfusion resulted in a comparable and substantial degree of cardiac remodeling but with a lower risk of cardiac rupture in comparison with MI model. This feature makes it a feasible model for cardiac ischemia research. By fully characterizing risk of fatal cardiac rupture, cardiomyocyte fate, systemic and regional inflammatory response, myocardial tensile strength and underlying mechanism and cardiac remodeling, our study provided a wide vision and new understanding for murine ischemia models with or without reperfusion. This work holds constructive and scientific values for other researchers in selection of proper animal models.

## Supporting information

S1 Fig Correlation between left ventricle (LV) weight and LV mass by echocardiography at 4 weeks after surgery. (a) Early reperfusion group (1h and 4h I/R groups). (b) Delayed reperfusion group (12h and 24h I/R groups). (c) MI group.
(DOCX)

S1 Table Echocardiographic analysis from the short-axis images (M-mode) at different time points after surgical interventions. (DOCX)

S2 Table Echocardiographic analysis from the long-axis (B-mode) at different time points after surgical interventions. (DOCX)

S1 Raw Images Raw images of Fig 4b. (PDF)

S1 Data Ct values of housekeeping related to Fig 3a–f. (XLSX)
