# Supplementary material for: Ischemic duration determines extent of cardiac remodeling, and both early and delayed reperfusion prevent fatal cardiac rupture: Model comparison
Source: PLoS One. 2025 Aug 22;20(8):e0328001. doi: 10.1371/journal.pone.0328001 (PMC12373173; doi:10.1371/journal.pone.0328001)
Supplement: S1 Fig — (a) Early reperfusion group (1h and 4h I/R groups). (b) Delayed reperfusion group (12h and 24h I/R groups). (c) MI group. (DOCX) [file pone.0328001.s001.docx]

**PONE-D-24-55939 R1**

**Ischemic duration determines extent of cardiac remodeling, and both early and** **delayed** **reperfusion** **prevent fatal cardiac rupture: model comparison**

Ling Zhao^1,2^^¶^, Amanguli Ruze^1,2¶^, Guo-Li Du^1,2,3^, Min-Tao Gai^1,2,4^, Jing Tang^1,2,6^, Xiao-Ming Gao^1,2,4*^

*^1^ State Key Laboratory of Pathogenesis, Prevention and Treatment of High Incidence Diseases in Central Asia, Department of Cardiology,* *First Affiliated Hospital, Clinical Medical Research Institute* *of Xinjiang Medical University, Urumqi, China*

*^2^ Xinjiang Key Laboratory of Medical Animal Model Research, Urumqi, China*

*^3^ Department of Endocrinology, First Affiliated Hospital of Xinjiang Medical University, Urumqi, China*

*^4^ Xinjiang Key Laboratory of Cardiovascular Disease, Urumqi, China*

*^5^ Department of Clinical Laboratory, First Affiliated Hospital of Xinjiang Medical University, Urumqi, China*

***Short title: Characteristics of ischemic heart models***

***Corresponding author**

Prof. Xiao-Ming Gao

State Key Laboratory of Pathogenesis, Prevention and Treatment of High Incidence Diseases in Central Asian, Clinical Medical Research Institute of Xinjiang Medical University.

137 Liyushan South Road, Urumqi, 830054, China.

Email: xiaoming.gao@xjmu.edu.cn

¶These authors contributed equally to this work.

**S1 Fig**


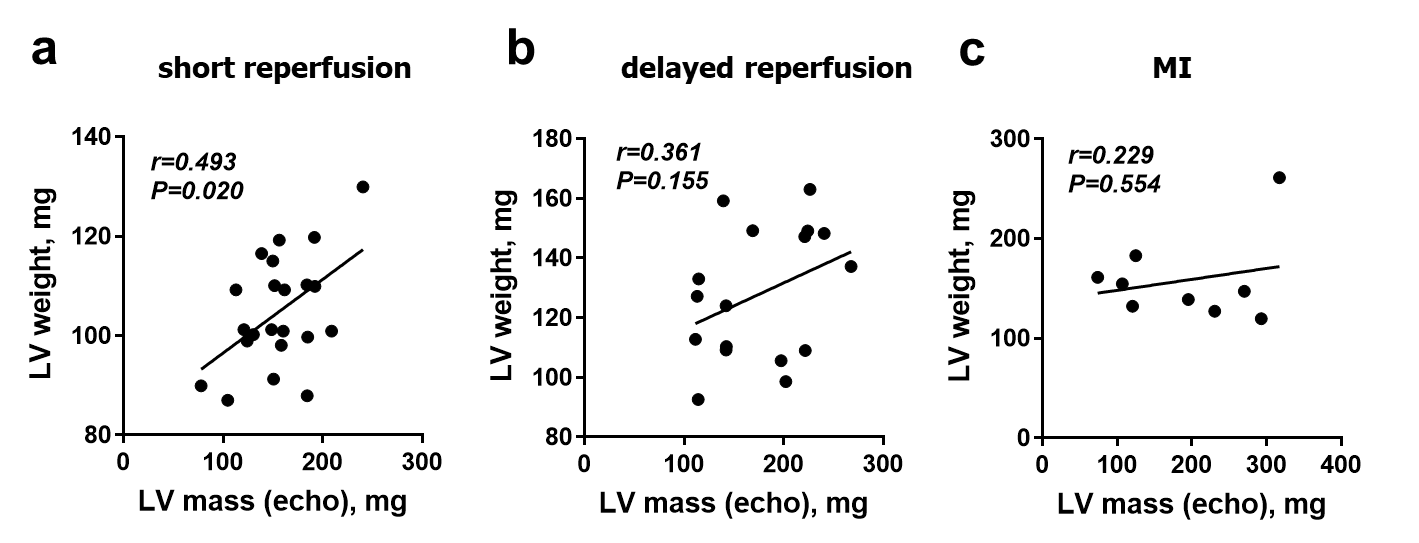


**S1 Fig. Correlation between left ventricle (LV) weight and LV mass by echocardiography at 4 weeks after surgery.** (**a**) Early reperfusion group (1h and 4h I/R groups). (b) Delayed reperfusion group (12h and 24h I/R groups). (c) MI group.
