# Supplementary material for: Ischemic duration determines extent of cardiac remodeling, and both early and delayed reperfusion prevent fatal cardiac rupture: Model comparison
Source: PLoS One. 2025 Aug 22;20(8):e0328001. doi: 10.1371/journal.pone.0328001 (PMC12373173; doi:10.1371/journal.pone.0328001)
Supplement: S2 Table — (DOCX) [file pone.0328001.s003.docx]

**PONE-D-24-55939 R1**

**Ischemic duration determines extent of cardiac remodeling, and both early and** **delayed** **reperfusion** **prevent fatal cardiac rupture: model comparison**

Ling Zhao^1,2^^¶^, Amanguli Ruze^1,2¶^, Guo-Li Du^1,2,3^, Min-Tao Gai^1,2,4^, Jing Tang^1,2,6^, Xiao-Ming Gao^1,2,4*^

*^1^ State Key Laboratory of Pathogenesis, Prevention and Treatment of High Incidence Diseases in Central Asia, Department of Cardiology,* *First Affiliated Hospital, Clinical Medical Research Institute* *of Xinjiang Medical University, Urumqi, China*

*^2^ Xinjiang Key Laboratory of Medical Animal Model Research, Urumqi, China*

*^3^ Department of Endocrinology, First Affiliated Hospital of Xinjiang Medical University, Urumqi, China*

*^4^ Xinjiang Key Laboratory of Cardiovascular Disease, Urumqi, China*

*^5^ Department of Clinical Laboratory, First Affiliated Hospital of Xinjiang Medical University, Urumqi, China*

***Short title: Characteristics of ischemic heart models***

***Corresponding author**

Prof. Xiao-Ming Gao

State Key Laboratory of Pathogenesis, Prevention and Treatment of High Incidence Diseases in Central Asian, Clinical Medical Research Institute of Xinjiang Medical University.

137 Liyushan South Road, Urumqi, 830054, China.

Email: xiaoming.gao@xjmu.edu.cn

¶These authors contributed equally to this work.

**S2 Table.** **Echocardiographic analysis from the long-axis (B-mode) at different time points after surgical interventions.**

| Variables  (B-mode) | Baseline  (n=9) |  | Week-1 | | | | |  | Week-4 | | | | |
| --- | --- | --- | --- | --- | --- | --- | --- | --- | --- | --- | --- | --- | --- |
|  |  |  | 1h I/R  (n=12) | 4h I/R  (n=10) | 12h I/R  (n=9) | 24h I/R  (n=9) | MI  (n=10) |  | 1h I/R  (n=12) | 4h I/R  (n=10) | 12h I/R  (n=9) | 24h I/R  (n=8) | MI  (n=9) |
| LVESA, mm^2^ | 17.8±1.6 |  | 22.4±0.3* | 24.3±0.3* | 26.7±0.6*† | 29.6±0.5*†‡ | 30.7±0.5*†‡ψ |  | 26.1±0.4*§ | 26.1±0.4* | 27.5±1.4* | 35.3±0.6*†‡ψ§ | 37.6±1.4*†‡ψ§ |
| LVEDA, mm^2^ | 27.9±1.2 |  | 28.7±0.2 | 30.4±0.4 | 31.3±0.6 | 33.6±0.5* | 34.5±0.4*† |  | 32.1±0.5 | 33.1±0.4 | 33.8±1.0* | 38.9±0.7*† | 41.6±1.2*†‡ψ§ |
| LVESV, μl | 43.1±5.8 |  | 65.9±1.2 | 75.4±1.8* | 86.0±3.3*† | 96.5±2.8*†‡ | 112.9±2.4*†‡ψ |  | 86.3±2.2*§ | 89.3±2.2* | 100.1±7.9* | 132.5±4.1# | 176.5±8.3# |
| LVEDV, μl | 85.9±6.2 |  | 97.3±1.2 | 105.8±2.1* | 108.4±3.8* | 116.0±2.9*† | 128.3±2.5*†‡ψ |  | 120.2±2.8*§ | 121.9±2.5* | 125.7±6.9* | 156±5.5#§ | 199.3±7.2#§ |
| EF, % | 50.1±5.2 |  | 34.1±0.7* | 28.8±0.7* | 27.0±2.9* | 17.3±0.4*† | 15.0±1.5*†‡ |  | 28.3±0.6* | 27.2±0.9* | 21.7±0.9* | 14.9±0.5*† | 11.8±0.9*† |
| SV, μl | 42.7±5.0 |  | 31.4±0.5 | 30.4±1.0 | 22.5±1.1* | 19.5±0.4*† | 15.4±1.1*†‡ |  | 33.7±1.0 | 32.7±1.0 | 25.5±2.1* | 24.4±1.9* | 22.8±1.7* |
| CO, ml/min | 19.7±2.0 |  | 15.3±0.3* | 13.8±0.4* | 9.4±0.7*†‡ | 9.2±0.3*†‡ | 7.1±0.5*†‡ |  | 15.2±0.4* | 14.4±0.4* | 12.0±0.9*† | 11.4±0.9*† | 11.1±0.9*†‡§ |

Values are mean ± SEM. 1h I/R, 4h I/R, 12h I/R, 24h I/R, mice subjected to 1h, 4h, 12h or 24h ischemia followed by 1 week or 4 weeks reperfusion; MI, myocardial infarction; LVESA, left ventricular end-systolic area; LVEDA, LV end-diastolic area; LVESV, LV end-systolic volume; LVEDV, LV end-diastolic volume; EF, ejection fraction; SV, stroke volume; CO, cardiac output. **P*<*0.05* vs. baseline. †*P<0.05* vs. 1h I/R at the same time point. ‡*P<0.05* vs. 4h I/R at the same time point. ψ*P<0.05* vs. 12h I/R at the same time point. #*P*<*0.05* vs. all other groups at the same time point. §*P<0.05* vs. week-1 at same group.
