# Supplementary figures and images for: Ischemic duration determines extent of cardiac remodeling, and both early and delayed reperfusion prevent fatal cardiac rupture: Model comparison
Source: PLoS One. 2025 Aug 22;20(8):e0328001. doi: 10.1371/journal.pone.0328001 (PMC12373173; doi:10.1371/journal.pone.0328001)

Figure 4b image

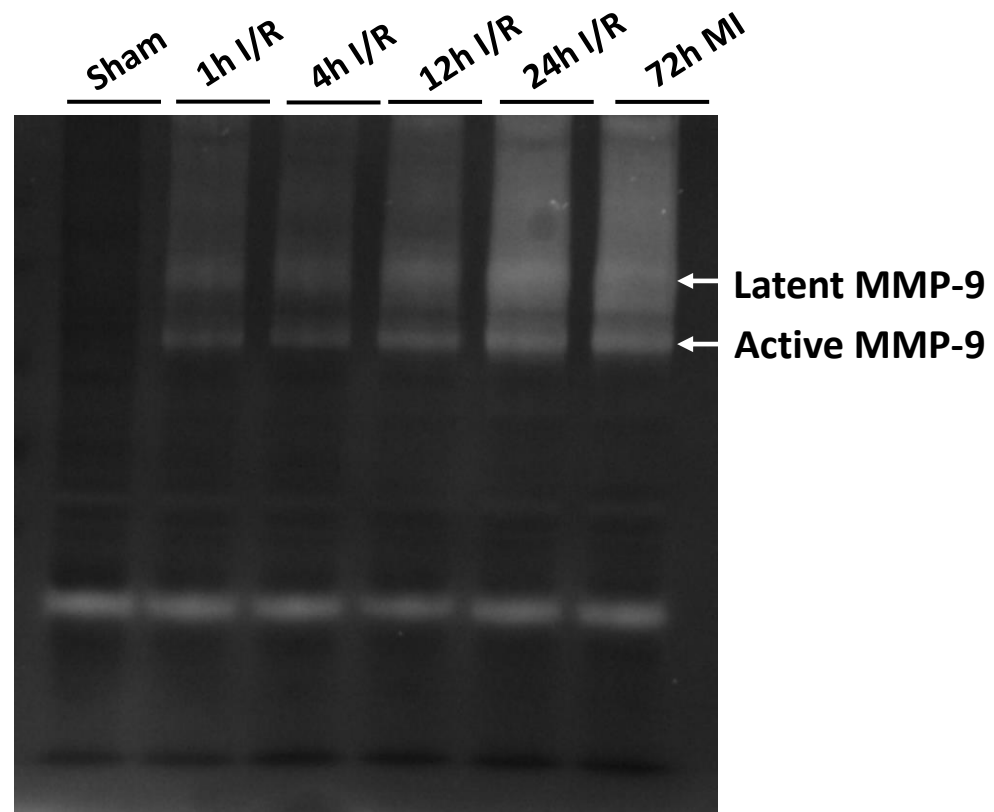

Supplement: S1 Raw Images — (PDF) [file pone.0328001.s004.pdf]
